# Quantification of the role of lead foil in flattening filter free beam reference dosimetry

**DOI:** 10.1002/acm2.13960

**Published:** 2023-03-13

**Authors:** Song Gao, Christopher Nelson, Congjun Wang, Vindu Kathriarachchi, Michael Choi, Rishik Saxena, Robin Kendall, Peter Balter

**Affiliations:** ^1^ Department of Radiation Physics The University of Texas MD Anderson Cancer Center Houston Texas USA

**Keywords:** beam quality, flattening filter free (FFF) beams, lead foil, TG‐51 addendum protocol

## Abstract

**Purpose:**

To quantify the potential error in outputs for flattening filter free (FFF) beams associated with use of a lead foil in beam quality determination per the addendum protocol for TG‐51, we examined differences in measurements of the beam quality conversion factor k_Q_ when using or not using lead foil.

**Methods:**

Two FFF beams, a 6 MV FFF and a 10 MV FFF, were calibrated on eight Varian TrueBeams and two Elekta Versa HD linear accelerators (linacs) according to the TG‐51 addendum protocol by using Farmer ionization chambers [TN 30013 (PTW) and SNC600c (Sun Nuclear)] with traceable absorbed dose‐to‐water calibrations. In determining k_Q_, the percentage depth‐dose at 10 cm [PDD(10)] was measured with 10×10 cm^2^ field size at 100 cm source‐to‐surface distance (SSD). PDD(10) values were measured either with a 1 mm lead foil positioned in the path of the beam [%dd(10)_Pb_] or with omission of a lead foil [%dd(10)]. The %dd(10)x values were then calculated and the k_Q_ factors determined by using the empirical fit equation in the TG‐51 addendum for the PTW 30013 chambers. A similar equation was used to calculate k_Q_ for the SNC600c chamber, with the fitting parameters taken from a very recent Monte Carlo study. The differences in k_Q_ factors were compared for with lead foil vs. without lead foil.

**Results:**

Differences in %dd(10)x with lead foil and with omission of lead foil were 0.9 ± 0.2% for the 6 MV FFF beam and 0.6 ± 0.1% for the 10 MV FFF beam. Differences in k_Q_ values with lead foil and with omission of lead foil were −0.1 ± 0.02% for the 6 MV FFF and −0.1 ± 0.01% for the 10 MV FFF beams.

**Conclusion:**

With evaluation of the lead foil role in determination of the k_Q_ factor for FFF beams. Our results suggest that the omission of lead foil introduces approximately 0.1% of error for reference dosimetry of FFF beams on both TrueBeam and Versa platforms.

## 1 INTRODUCTION

Flattening filter free (FFF) beams have become common in linear accelerator (linac)‐based radiotherapy. Because the flattening filter is removed from the beam path, the FFF beams have higher dose per pulse (DPP) and more forward peaked bremsstrahlung than the flattened beam. The process for measuring absolute dose for flattened beams has been outlined in the AAPM TG‐51^1^ protocol. An addendum^2^ to TG‐51 includes guidance for FFF beams to address ionization recombination effects due to high DPP and volume averaging effects from unflattened beam profiles. Comprehensive evaluations of these two effects were previously published.^3^, ^4^ To determine the beam quality conversion factor k_Q_, TG‐51^1^ recommends using a 1 mm lead foil for beam energies higher than 10 MV to remove unknown electron contamination from the linac head. TG‐51 addendum^2^ no longer recommends the use of lead foil for flattened high‐energy photon beams. A comprehensive study^5^ showed that omitting lead foil led to errors in k_Q_ of no more than 0.2% and simplified the measurement procedure, making it less prone to operator error. The TG‐51 addendum^2^ still recommends use of a 1 mm lead foil for FFF beams owing to a lack of data. A comprehensive comparison between recommendations of the TG‐51 and TG‐51 addendum reference dosimetry protocols^4^ demonstrated that differences in k_Q_ determined using the two protocols for PTW N30013 ionization chambers were within 0.1% for FFF beams. For the TG‐51 addendum protocol, they also considered corrections for defined radiation field size, ion chamber leakage, and volume averaging effects in the output measurements. Another investigation of the effect of including lead foil in the beam quality measurement for 6 MV FFF and 10 MV FFF beams^6^ showed that the change in k_Q_ was 0.1% on a Varian TrueBeam linac for the PTW 30013 Farmer chamber. That group included ion recombination and beam non‐uniformity corrections in percent depth dose at 10 cm, PDD(10), measurements obtained with lead foil, but those two corrections have not been considered for PDD(10) measurements with the omission of lead foil. A very recent publication of guidance for TG‐51 reference dosimetry^7^ recommends that the use of lead foil is required for high accuracy determination of beam quality specifiers for photon beams with energy of 10 MV and higher based on the previously studies.^5^

The purpose of this study was to quantify the differences in the beam quality conversion factor k_Q_ between the use of lead foil and with omission of use lead foil for FFF beams under the same measurement conditions. From these differences in k_Q_, measured on several TrueBeam and Versa HD linacs, the error in output resulting from omitting the lead foil was determined.

## 2 METHODS AND MATERIALS

**FIGURE 1:**
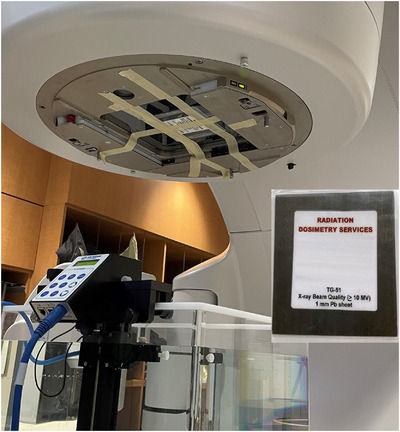
The setup for beam quality measurements involving lead foil.

We measured the output a 6 MV FFF and a 10 MV FFF beam on eight Varian TrueBeam linacs (Varian Medical Systems, Palo Alto, California, USA), and two Elekta Versa HD linacs (Elekta Solutions AB, Stockholm, Sweden). The measurements were conducted in a 1D water tank by using Farmer ionization chambers with traceable absorbed dose‐to‐water calibrations and PC electrometer (Sun Nuclear, Melbourne FL), also with traceable calibrations. Two types of waterproof Farmer ionization chambers were used, a PTW 30013 with an active volume of 0.6 cm^3^ (PTW, Freiburg, Germany) and an SNC600c also with an active volume of 0.6 cm^3^ (Sun Nuclear, Melbourne FL). The PDD(10) measurements were made both with a lead foil and without a lead foil in place. All PDD(10) measurements were done with a 10 × 10 cm^2^ field size at 100 cm source‐to‐surface distance (SSD), and the data was shifted to the effective point of measurement of the ion chamber as specified in the TG‐51 report.^1^ The measurements with the lead foil [%dd(10)_Pb_] and without the lead foil [%dd(10)] were acquired under the same setup conditions. When lead foil was used, it was placed in the exit window of the machine head, approximately 50 cm from the water surface (Figure 1).

When the lead foil was used, the photon component of the PDD(10), *%dd(10)_x_*, was calculated from the %dd(10)_Pb_ according to eq. (13) in TG‐51^1^:

$$\begin{array}{ccc} \;\%dd{\left(10\right)}_{x} & = & \;\left[0.8905+0.00150\cdot \%dd{\left(10\right)}_{Pb}\right]\\ & & \cdot \%dd{\left(10\right)}_{Pb}, \end{array}$$

if $\%dd{\left(10\right)}_{Pb}\geq 73\%;\;$otherwise, $\%dd{\left(10\right)}_{x}=\;\%dd{\left(10\right)}_{Pb}$.

When lead foil was omitted, *%dd(10)_x_*, was determined directly from %dd(10), $\%dd{\left(10\right)}_{x}=\;\%dd\left(10\right)$, for both 6 MV FFF and 10 MV FFF beams. It should be noted that for the 10 MV FFF beam on the Versa in this study the %dd(10)_Pb_ required the use of the above empirical formula but the %dd(10) was below the threshold required for the use of the interim alternative formula in TG‐51.^1^ The differences in *%dd(10)_x_* with and without lead foil were compared for the measurement data from the ten linacs.

The beam quality correction factor k_Q_ then was determined by using the empirical fit equation (1) in the TG‐51 addendum^2^:

$$\begin{array}{ccc} k_{Q} & = & a+b\cdot 10^{-3}\cdot \left(\%dd{\left(10\right)}_{x}\right)\\ & & +\;c\cdot 10^{-5}\cdot {\left(\%dd{\left(10\right)}_{x}\right)}^{2}, \end{array}$$

$$\;63<\;\%dd{\left(10\right)}_{x}<86.$$

The fitting parameters, a = 0.9562, b = 2.141, and c = −2.623, for the PTW 30013 ionization chamber were from Table 1 in the TG‐51 addendum,^2^ whereas the fitting parameters for the SNC600c ionization chamber were from Table 6 of the recent Monte Carlo study.^8^ The SNC600c ionization chamber was used for Linac 1 and Linac 2, and the PTW 30013 ionization chamber were used for the other linacs. Two metrics were chosen to compare the effects of measurements with lead foil and with the omission of lead foil, %dd(10)_x_ and k_Q. ._ The change in %dd(10)_x_ (with foil) was quantified by *Δ[%dd(10)_x_] = %dd(10)_x_[lead]—%dd(10)_x_[no lead]*; and the change in k_Q_ by *Δk_Q_ = k_Q_[lead]/ k_Q_[no lead]−1* for measurement data from 10 linacs. The output error due to the differences in k_Q_ factors was thus analyzed.

**TABLE 1:** Effect of using lead foil on 6 MV FFF and 10 MV FFF beam quality measurements

Beam/Linac	No Lead used			1 mm lead foil used			Difference (%)	
6 FFF	%dd(10)	%dd(10)_X_	k_Q_	%dd(10)_Pb_	%dd(10)_X_	k_Q_	Δ[%dd(10)_x_]	Δk_Q_
TrueBeam	63.39%	63.39%	0.9955	64.23%	64.23%	0.9945	0.8%	−0.10%
Versa	67.35%	67.35%	0.9904	68.00%	68.00%	0.9895	0.6%	−0.09%
** 10 FFF**	%dd(10)	%dd(10)_X_	k_Q_	%dd(10)_Pb_	%dd(10)_X_	k_Q_	Δ[%dd(10)_x_]	Δk_Q_
TrueBeam	71.01%	71.01%	0.9850	71.48%	71.48%	0.9842	0.5%	−0.08%
Versa	72.61%	72.61%	0.9824	73.15%	73.17%	0.9814	0.6%	−0.09%

The measured %dd(10) and the resultant %dd(10)_X_, and k_Q_ with omission of use lead foil were compared with those measurements made with a 1‐mm lead foil for the two individual linacs as an example.

## 3 RESULTS

**FIGURE 2:**
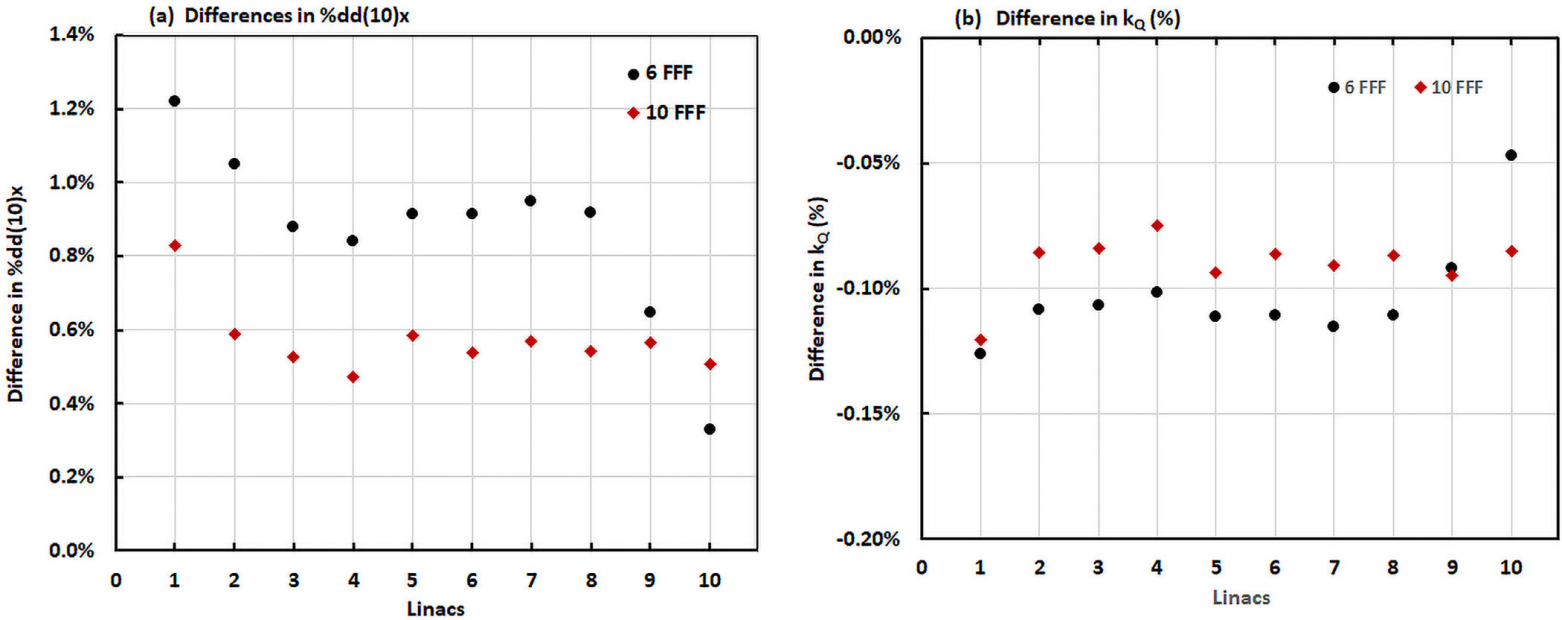
Effect of using lead foil on 6 MV FFF and 10 MV FFF beam quality measurements on 10 linacs. (a) Differences in %dd(10)x for no lead foil vs with lead foil. (b) Differences in the k_Q_ factor for no lead foil versus with lead foil. Linacs #1 and #2 are TrueBeam linacs measured with an SNC600c ionization chamber. Linacs #9 and #10 are Versa HD linacs measured with a PTW ionization chamber. The others linacs are TrueBeam machines measured with PTW ionization chambers.

Measurement results of %dd(10), %dd(10)_Pb_, and the beam quality parameters %dd(10)_x_, k_Q_, Δ[%dd(10)_x_], and Δk_Q_ for two individual linacs are shown in Table 1. The effect of including the lead foil in the beam quality measurement %dd(10)_x_, and the k_Q_ factors for all 10 linacs in this study (Figure 2a), indicate that the average differences in %dd(10)_x_ measured with lead foil and with omission of lead foil were 0.9 ± 0.2% for the 6 MV FFF beam and 0.6 ± 0.1% for the 10 MV FFF beam. The maximum differences in %dd(10)_x_ with lead foil and with omission of lead foil were 1.2% for the 6 MV FFF beam and 0.8% for the 10 MV FFF beam. When lead foil was used, the %dd(10)x value was always higher than that without lead foil for the same energy beam, because the electron contamination is reduced by using the lead foil, which is more pronounced at d_max_ than that at 10 cm depth.

Measurement data for all 10 linacs in this study (Figure 2b) also showed that the average differences in k_Q_ factor measured with and without lead foil were −0.1 ± 0.02% with a maximum of −0.13% for the 6 MV FFF beam, and −0.1 ± 0.01% with a maximum of −0.12% for the 10 MV FFF beam. Those results demonstrated that when calibrating FFF beams, the added uncertainty from the omission of using lead foil in the beam quality determinations is about 0.1%. Therefore, the error in output determination resulting from omitting of using lead foil is also about 0.1%.

## 4 DISCUSSION

To correct for electron contamination in photon beams at d_max_ during reference dosimetry, Li and Rogers concluded from Monte Carlo studies that placing a 1‐mm lead foil directly below the linac head yielded the best results.^9^, ^10^ The AAPM TG‐51 protocol recommends using 1 mm lead foil for determining the photon beam‐quality specifier %dd(10)_X_ for high‐energy beams above 10 MV, with the lead foil placed at 50  or 30 cm from the water surface. The AAPM TG‐51 addendum recommended using 1 mm lead foil only for FFF beams for all beam energies.^2^ The k_Q_ results we obtained for 6 MV FFF and 10 MV FFF beams from Varian TrueBeam and Elekta Versa HD linacs showed no significant differences for determining k_Q_ when Farmer‐type ionization chambers were used. Similar studies on Halcyon linacs for 6 MV FFF beams also indicated that machine output values differed by only 0.1% between the beam quality measurements without versus with 1 mm lead foil.^11^, ^12^ Our results were consistent with previous studies showing that the change in k_Q_ from including lead foil in the beam was sufficiently small compared with the beam quality data obtained without lead foil in the beam for reference dosimetry of both 6 MV FFF and 10 MV FFF beams on TrueBeam and Versa platforms with Farmer‐type ionization chambers.

We did not include the effect of the variation in P_ion_ between d_max_ and 10 cm depth in k_Q_ determination. The variation of P_ion_ with depth is chamber dependent and is larger for FFF beams than for flattened beams. We found that for the PTW 30013 (Farmer) chamber the variation of P_ion_ between d_max_ and 10 cm was 0.3% in 6 MV FFF beam and 0.2% in 10 MV FFF beam, which is consistent with previous studies.^13^ The P_ion_ variation of this chamber adds a maximum 0.03% uncertainty in k_Q_ due to the shallow slope of the k_Q_ versus PDD(10)_X_ curve. This result may not apply for other type chambers and/or bias voltages and should be studied by each user with their equipment.

## 5 CONCLUSION

The recommendations of the TG‐51 guidances^2^, ^7^ require the use of lead foil in k_Q_ determination of FFF beam reference dosimetry. Our results of 6 MV FFF and 10 MV FFF beams from both TrueBeam and Versa linacs suggest that a 0.1% of error introduced in FFF beam reference dosimetry from the omission of lead foil.

## AUTHOR CONTRIBUTIONS

Christopher Nelson and Peter Balter: designed the study and edited the manuscript.

Song Gao: designed the study, collected measurement data, analyzed results, wrote and edited the manuscript.

Congjun Wang: collected measurement data, analyzed results, and edited the manuscript.

Vindu Kathriarachchi, Michael Choi, Rishik Saxena, Robin Kendall: collected measurement data and edited the manuscript.

## CONFLICT OF INTEREST

None.

## Data Availability

The data that support the findings of this study are available from the corresponding author upon reasonable request.
